# Unusual Lignocellulosic Bioresins: Adhesives and Coatings for Metals and Glass

**DOI:** 10.3390/molecules29225401

**Published:** 2024-11-15

**Authors:** Antonio Pizzi

**Affiliations:** Laboratoire d’Etude et de Recherche sur le Materiau Bois, University of Lorraine, 27 Rue Philippe Seguin, 88000 Epinal, France; antonio.pizzi@univ-lorraine.fr

**Keywords:** tannin, lignin, citric acid, glucose, non-isocyanate polyurethanes, bio-sourced adhesives, bio-sourced coatings, metals, glass, Teflon

## Abstract

This minireview presents some unusual but encouraging examples of lignocellulosic-based adhesives and coatings used for metals, glass, and some other difficult-to-adhere materials. The reactions and applications presented are as follows. (i) The reactions of tannins and wood lignin with phosphate salts, in particular triethylphosphate, to adhere and join steel and aluminum to Teflon, in particular for non-stick frying pans. These adhesive coatings have been shown to sustain the relevant factory industrial test of 410 °C for 11 min and, moreover, to present a 50% material loss even at 900 °C for 5 min. (ii) Non-isocyanate polyurethanes (NIPU) based on glucose and sucrose as coatings of steel and glass. These were obtained by the carbonation of carbohydrates through reaction with the inexpensive dimethyl carbonate followed by reaction with a diamine; all materials used were bio-sourced. Lastly, (iii) the use of citric acid-based adhesive coupled with any hydroxyl groups carrying material for coating metals is also described. These three approaches give a clear indication of the possibilities and capabilities of biomaterials in this field. All these are presented and discussed.

## 1. Introduction

Mainly general bio-sourced resin reviews exist, although often, they are mainly aimed at clarifying the nature of the polymers formed rather than emphasizing the possibility of these biopolymers for adhesives, coatings, and bonding applications [1]. Bio-sourced resins of all sorts have been developed mainly to bond and coat wood, and in these specific cases, such resins are tested for adhesives and even used industrially [2,3,4,5,6,7,8]. Sometimes, among the types of resins developed, some of them show unusually good properties for bonding and coating more difficult substrates than wood, such as metals and even glass. Lignocellulosic-based bio-adhesives to bond metals and glass have already been reported and are mainly based on alginates [9]. There are, however, many other articles dealing with different biomaterials used for bioadhesives for metals, glass, and silicates for a number of applications as well as lignocellulosic materials [10,11,12,13,14,15,16,17,18,19,20,21,22,23,24] and even some reviews on the same subject [25,26]. The literature used in this study was gathered from the database of Google Scholar using the keywords complete bioresins for metals and glass considering the period between 2014 and 2024. This minireview presents some metal binding adhesive cases based on different bio-sourced lignocellulosic materials.

## 2. Tannin Triethyl Phosphate for Biobinders for Metal/Teflon Assemblies

Reacting triethylphosphate (TEP) with flavonoid tannins yields a thermally resistant adhesive capable of binding Teflon to metals like steel and aluminum [27].

**Figure 1:**
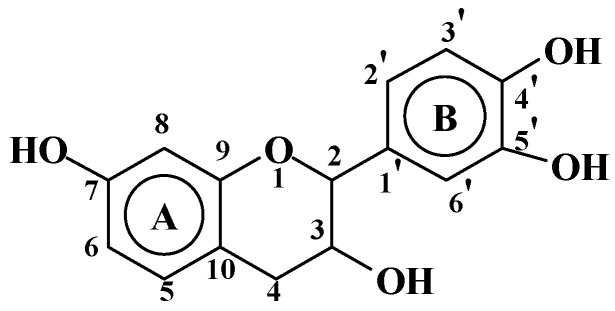
Structure of a flavonoid unit with atom numbering.

**Figure 2:**
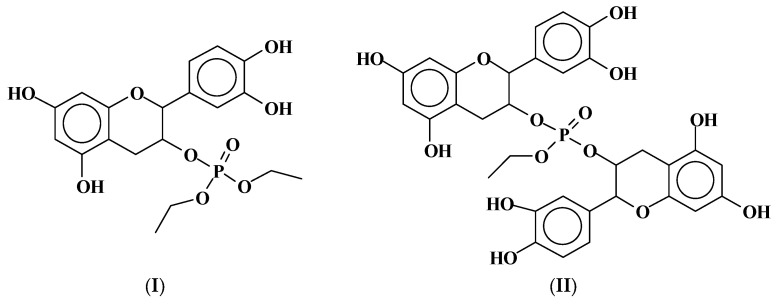
Basic structures (**I**) and (**II**) produced by the reaction of triethylphosphate on the C3 site of tannin flavonoid units [27].

On the flavonoid unit structure (Figure 1) the reaction with TEP occurs at temperatures in the 160–185 °C range and leads to the formation of several structures, e.g., Figure 2.

The reaction appears to take place first with the flavonoid units’ –C_3_ –OH groups that have alcoholic behavior. This site appears to be the favorite TEP reaction site on flavonoid tannin units, and it is extensive. It must be pointed out that the reaction seems to have a temperature of activation below which it does not appear to occur. Thus, it occurs readily at 185 °C but does not occur at 100 °C.

**Figure 3:**
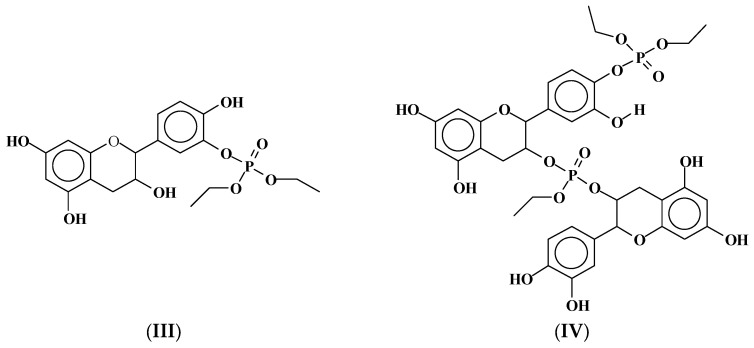
Basic structures (**III**) and (**IV**) produced by the reaction of triethylphosphate at the aromatic B-ring C4′ and C5′ sites of tannin flavonoid units, coupled with the reaction at the C3 site [27].

A lower level of the TEP reaction also takes place on the phenolic –OH groups on the flavonoid unit’s B-ring C4′ and C5′ sites, as shown by the detected structures in Figure 3.

**Figure 4:**
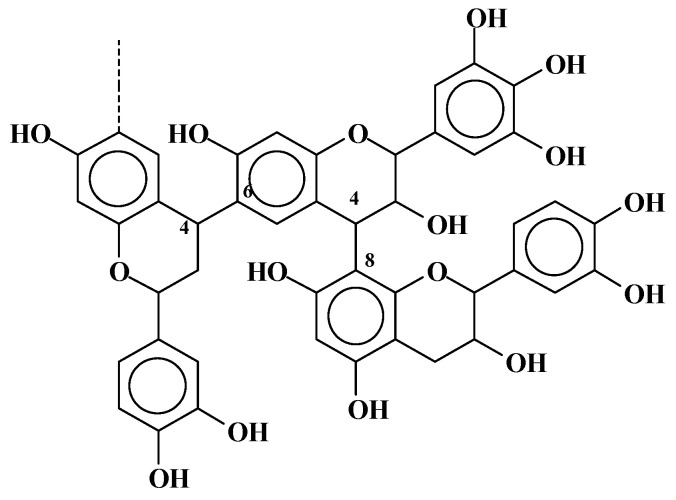
Structure of a trimer of mimosa tannin showing C4–C8 linkage between the terminal and before-terminal flavonoid unit and C4–C6 linkage between the before-terminal flavonoid unit and all subsequent flavonoid units [2].

Mimosa tannin was used in this research work. It is composed of catechin units (approximately 15%), robinetinidin units (approximately 65–70%), and fisetinidin units (approximately 15–20%), which are bonded to C4 to C6 in the tannin oligomers, with the exception of catechin, which is linked to C4 to C8 and which is, in general, placed as the lower terminal unit of the tannin oligomers. Mimosa trimers, for example, present a structure such as that shown in Figure 4.

This is reported to explain that structures I, II, III, and IV are then valid for both catechin as well as for robinetinidin in the tannin of the mimosa tannin; these two units present the same molecular weight.

**Figure 5:**
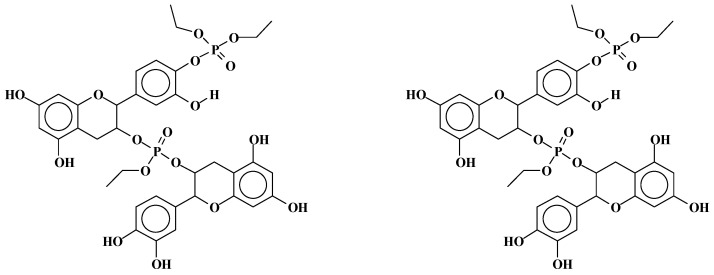

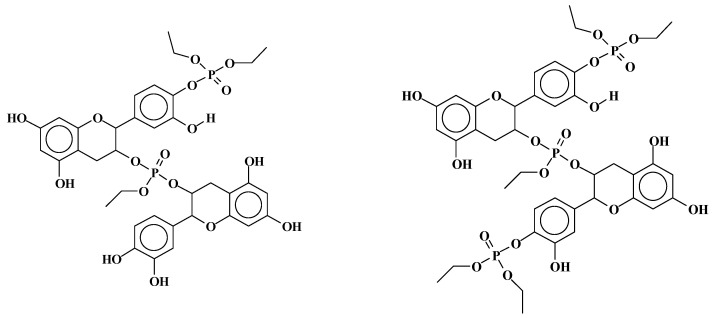
A variety of structures detected in the reaction of flavonoid tannins with triethylphosphate [27].

MALDI–ToF mass spectrometry confirmed the formation of the structures above and below with a note that a TEP reaction was not found to occur on the A-ring –OH groups but only on the B-ring ones, and the latter occurred as a lesser reaction pattern (Figure 5).

**Figure 6:**
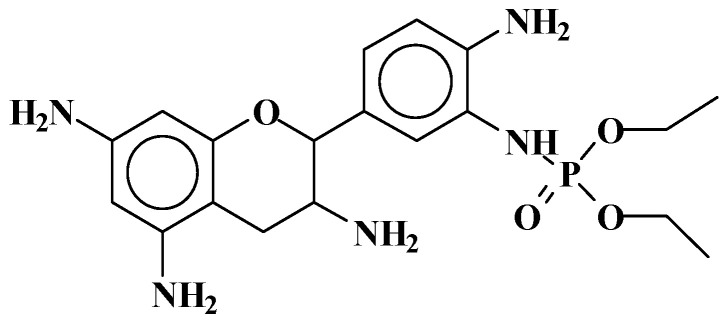
Structure of the reaction of triethylphosphate with an aminated flavonoid tannin unit [27].

The research work also reported that the formulation to which ammonia is added seems to favor the reaction of TEP with the tannin flavonoid unit. The amination of the hydroxyl groups of the tannin, both of phenolic and alcoholic nature, is well-known [28]. This appears to be due to the amination of some of the hydroxyl groups of the flavonoid to be transformed in –NH_2_ groups. The reaction of the aminated C3 of the aminated tannin detected by NMR was also confirmed by the MALDI ToF investigation, with species such as that shown in Figure 6 being detected.

**Figure 7:**
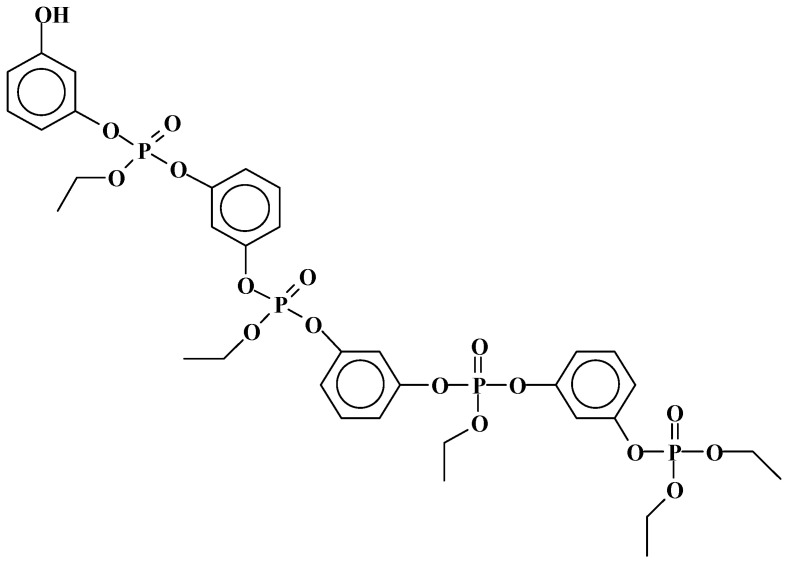
Higher-molecular-weight oligomer species detected by reaction of resorcinol used as a simple model compound of aromatic rings of tannin [27].

Moreover, the possibility of the formation of higher-molecular-weight oligomers was confirmed by MALDI ToF analysis by reacting TEP with resorcinol as a simple model compound of the A-ring of flavonoid units yielding higher-molecular-weight oligomers within the range examined, as shown in Figure 7.

**Figure 8:**
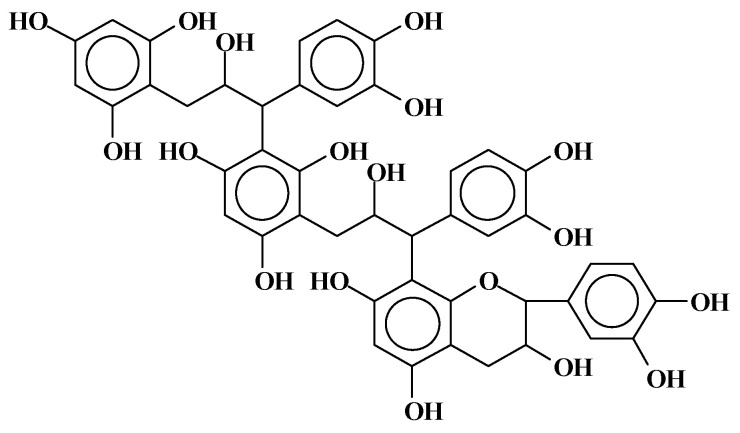
Structure of a flavonoid tannin trimer showing a number of flavonoid units where the heterocyclic ring of the structure has been cleaved and the structure is open [2].

Such model compounds’ approach indicates that while the C3-OH of flavonoid units is the favorite reaction site for TEP, followed by the phenolic –OHs on the flavonoid units B-ring, the possibility of the reaction of TEP on the flavonoid unit’s A-rings also exists, and it is likely to occur. This possibility was confirmed by the finding that in the ^13^C NMR analysis in this research work, it was found that a consistent number of catechin and/or robinetinidin units presented open heterocyclic rings of the flavonoid units, thus showing that phenolic A-rings effectively have a resorcinol or phloroglucinol nature once reaction with TEP was carried out, as shown in Figure 8.

The research work also tested the progression of the degradation of the tannin alone, tannin+NH_3_, tannin+TEP, and tannin+NH_3_+TEP up to 900 °C for a period of 120 min. The materials showed a multi-step degradation process. An initial weight loss step was detected mostly for tannin (~10%) and tannin+NH_3_ (~5%) in the 50–170 °C range due to the evaporation of absorbed water and volatiles (CO, H, and CO_2_). Indications of the higher thermal stability of the materials prepared using TEP emerged from this test. A second step of the thermal degradation for the tannin alone occurs at around 320 °C, while for tannin+NH_3_, tannin+TEP, and tannin+TEP+NH_3_, this was respectively observed at 370, 350, and 405 °C. For the tannin alone, the temperature at which 50% was decomposed was 520 °C. For tannin+NH_3_, it was 750 °C, and 50% decomposition of tannin+TEP and tannin+TEP+NH_3_ occurred after only 5 min at 900 °C. The article also compares the residual material after heating at 900 °C. After 57 min at 900 °C, the residual materials under an argon atmosphere for tannin, tannin+NH_3_, tannin+TEP, and tannin+TEP+NH_3_ were 17%, 37%, 38%, and 44%, respectively. Thus, a high thermal stability has been observed for resins prepared by reacting tannin with TEP. It is important to note that these adhesive coatings have been shown to sustain the relevant factory industrial test of 410 °C for 11 min [29,30,31].

Such a reaction of TEP with a tannin has been patented and industrially applied to bond Teflon on steel and/or aluminum [27,29]. This was carried out to manufacture non-stick frying pans without using the currently used synthetic, oil-derived phenol formaldehyde resin. It must be pointed out that such adhesive passed the relevant test for the application of non-stick frying pans, namely resisting heating at 410 °C for more than 11 min. It then constitutes a major “green” advance for non-stick frying pans, but its use can be extended to other metal bonding applications.

## 3. Lignin Triethyl Phosphate for Wood Surface Coatings and Bioadhesive for Metals/Teflon Assembly

Considering that the reaction of TEP with tannins is patented, a further development was carried out for the same application but using lignin to react with triethylphosphate, and the results obtained were also very encouraging; this latter work was not patented [32,33]. Thus, new thermosetting resins were obtained by reacting TEP with lignin to develop through a different lignocellulosic material new heat-resistant bio-sourced paints, lacquers, adhesives, coatings, and resins for numerous different applications and different surfaces [32,33]. Moreover, guaiacol (purity > 99%) and glycerol (purity > 99.5%) were also used as simple model compounds of lignin to clarify what occurs. Lignin and TEP appear to polycondense because of the TEP reacting with both the phenolic hydroxyl groups and the side chain aliphatic hydroxyls groups of lignin. Higher temperatures and the addition of ammonia favor the reaction. Model compounds, namely guaiacol and glycerol, were used initially to interpret the reaction of TEP with lignin’s aromatic and aliphatic parts. What was found with the model compounds was then confirmed by reacting a desulfurized softwood kraft lignin (BioChoice kraft lignin) with TEP.

**Figure 9:**
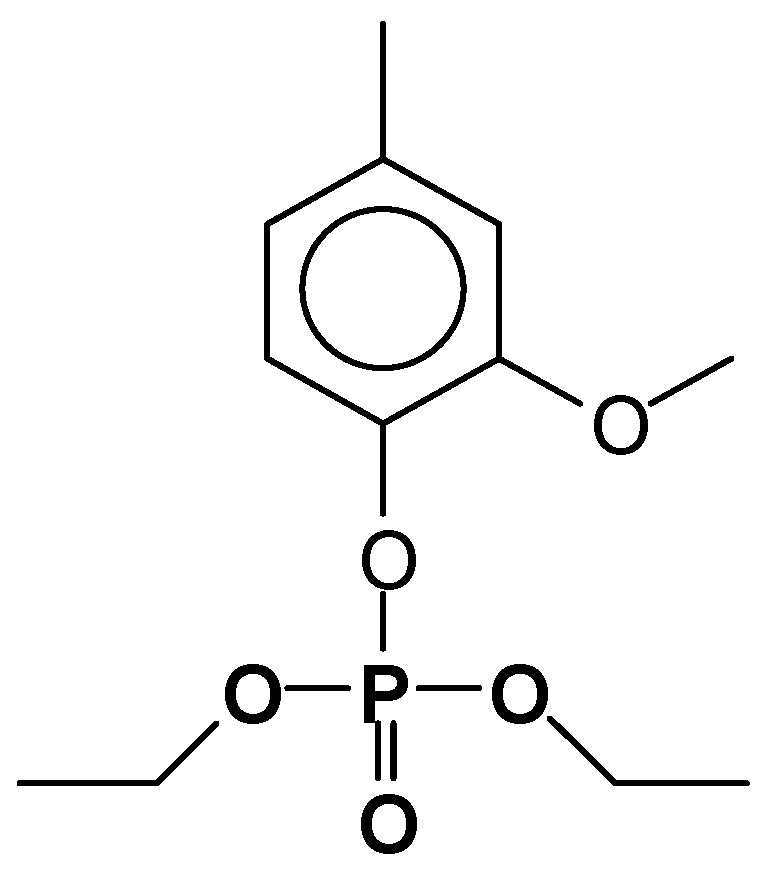
Example of TEP reaction site on lignin structure [32].

Acetone-insoluble hard, rigid, dark cured resin solids were obtained when reacting TEP and lignin at 180 °C and 220 °C. When TEP was reacted with glycerol at 180 °C, a transparent liquid resin was obtained, while when guaiacol was reacted with TEP without or with NH_3_ the resulting product was so burned that it was not possible to recover any resin solids after heat treatment. Finally, a flexible black semisolid was obtained when TEP and lignin were reacted at 90 °C; a resin soluble in acetone but not in water was obtained. The TEP heated at 90 °C with glycerol remained a transparent liquid before and after heating it. The resins prepared were analyzed by MALDI ToF, CP-MAS ^13^C NMR, and FTIR spectrometry. The NMR analysis showed, together with some unreacted lignin units, lignin units where TEP had reacted with the lignin phenolic –OH (Figure 9).

Also, the lignin aliphatic –CH_2_OH groups of the unit’s side chain appeared to react with TEP.

**Figure 10:**
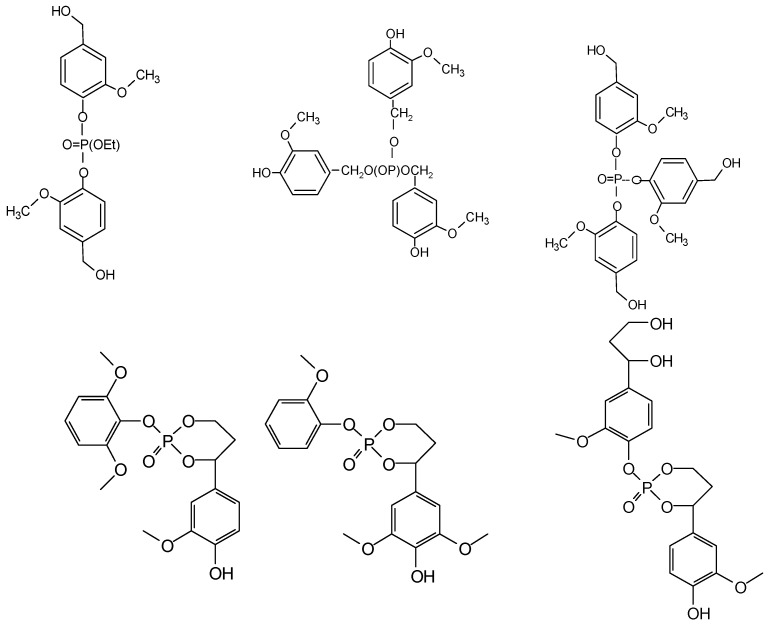
An example of the variety of structures formed by the reaction of triethyl phosphate with lignin units. Note also the cyclic structures formed by intramolecular reaction [32].

A variety of structures formed by the reaction were determined by combining ^13^C NMR and MALDI ToF analysis, such as those shown in Figure 10.

Branched and cyclic structures were also identified, the latter generated by intramolecular reactions, as shown in Figure 10.

Amination, in some cases by substitution with ammonia of the lignin phenolic –OH groups, also appeared to occur. This type of reaction occurs with ease and has been reported for other phenolic compounds [28].

**Figure 11:**
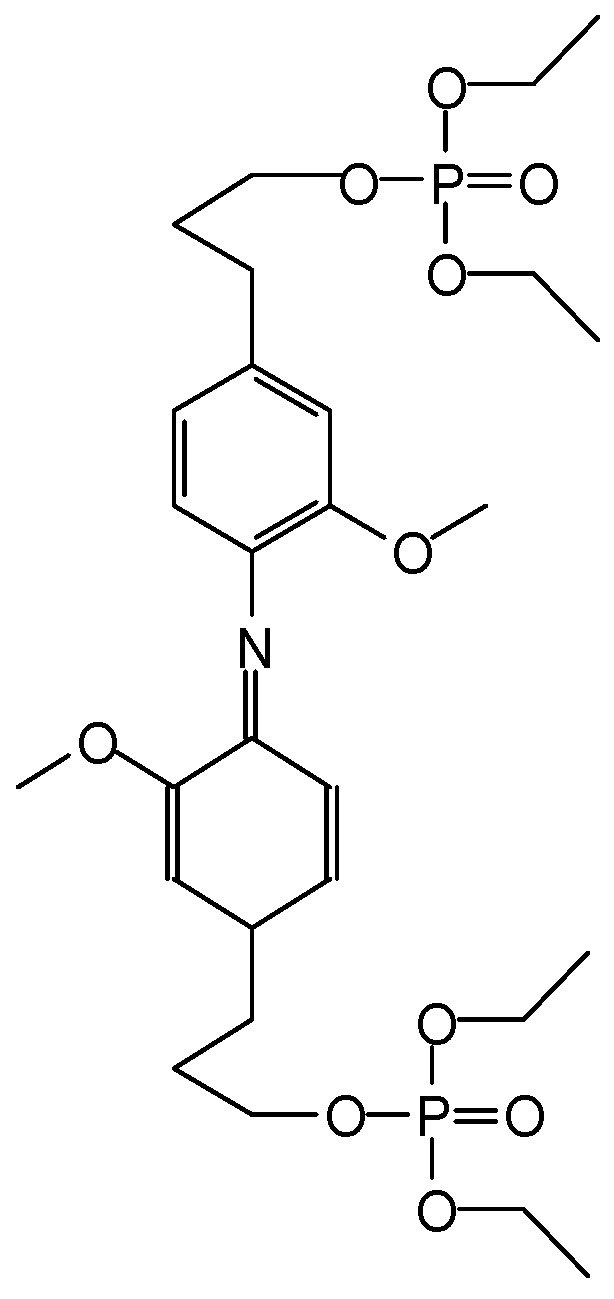
Example of a detected aminated lignin structure obtained by reaction with triethylphosphate [32].

All these reactions yielded characterized structures, as shown in Figure 11.

**Figure 12:**
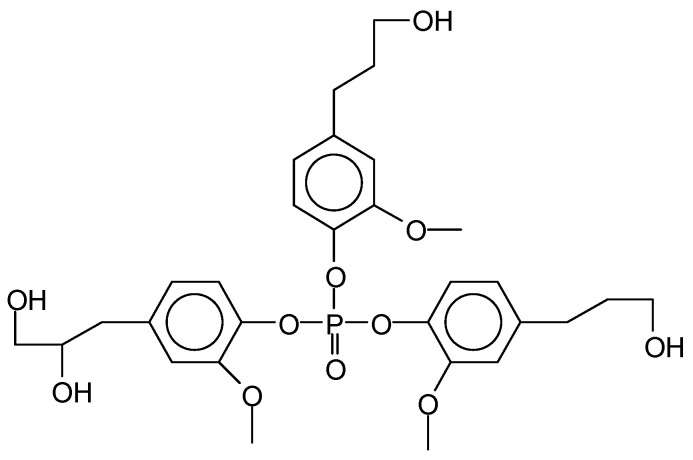
An example of a detected higher-molecular-weight oligomer produced by the reaction of triethylphosphate linking two lignin units [32]. Note: TEP can be linked through the phenolic –OH groups, as in the figure; with the aliphatic lignin –Ohs; or with both –OH types.

Finally, several indications of lignin demethylation reactions were noticed in the NMR analysis. Higher-molecular-weight oligomers of lignin units or their sequences reacted through TEP were also detected, such as that shown in Figure 12.

In Figure 12, the lignin units are linked through TEP. TEP can be reacted with either the phenolic or the aliphatic lignin unit’s sites.

**Figure 13:**
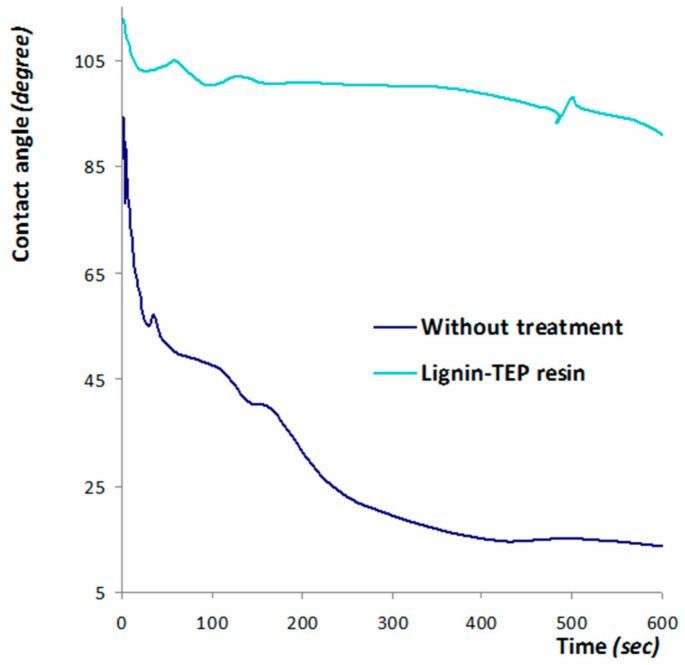

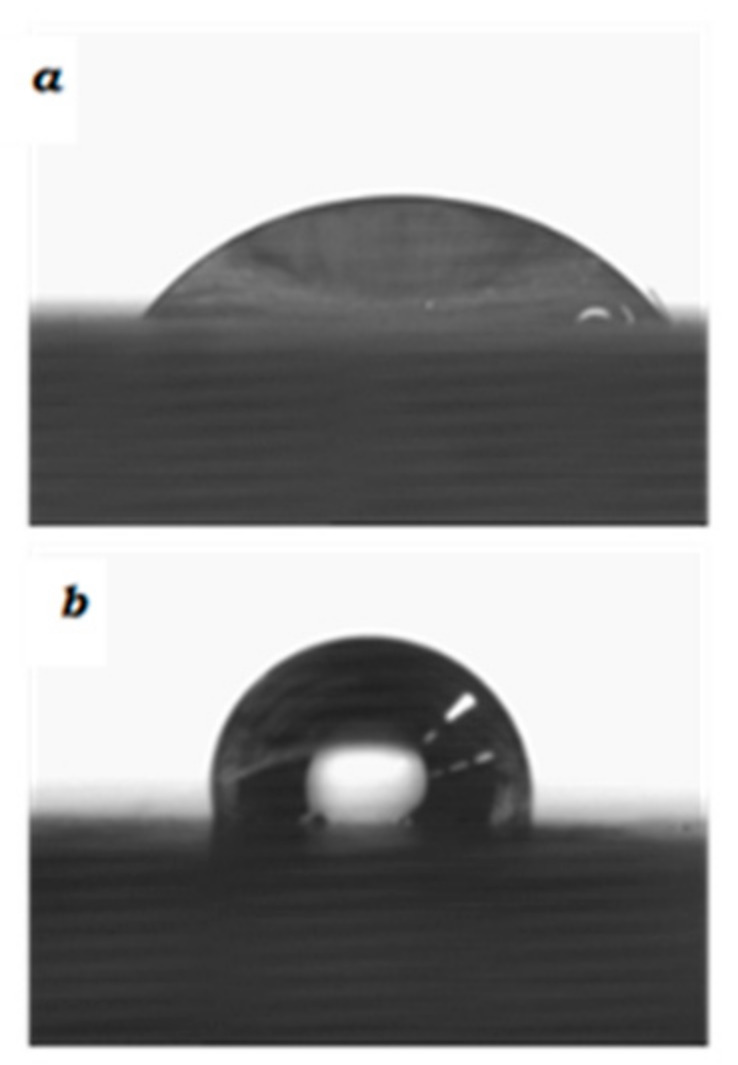
(**Top**) Water contact angle variation as a function of time of the lignin-TEP-based resin coated beech wood surface and the untreated beech sample control. (**Bottom**) Water drop shape after 60 s on: (**a**) untreated beech wood (control) and (**b**) beech wood surface coated with a lignin–TEP-based resin [32].

The effectiveness of the product yielded by the TEP reaction with lignin for surface coating applications on wood were measured by the dynamic sessile water drop test on a coated wood surface. Figure 13 shows the results obtained, confirming the effectiveness of the reacted product as a wood surface finish.

It is clear from Figure 13 that a much higher initial water contact angle is obtained for the TEP/lignin resin-coated wood surface than for the untreated wood surface. Furthermore, the water contact angle remains almost constant for the coated surface while showing a rapid time-dependent decrease on the untreated surface.

As for tannins, it appears that the TEP/lignin reaction is temperature-dependent, being favored from 180 °C. Hardened and insoluble resins are obtained, also with ammonia added, as additional cross-linking reactions do occur.

**Figure 14:**
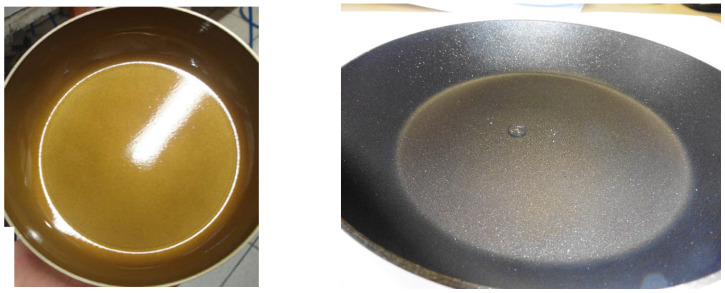
Frying pan metal base with the polyphenolic-TEP binder applied to it (**left**) and finished pan with Teflon applied on the binder according to a proprietary process (**right**) [33].

However, the reaction of lignin and tannins (and possibly other biopolymers) with TEP was initially developed for the specific use to bind Teflon on steel or aluminum for non-stick frying pans (Figure 14) [27,29].

This biopolyphenol–TEP adhesive has been used as a substitute for synthetic phenol-formaldehyde resins but also has the advantage of not using any formaldehyde. It needed to adhere well to both the non-stick frying pan metal base (steel and/or aluminum) and simultaneously to its Teflon coating. Figure 14 shows a polyphenol–TEP resin adhered to the metal base without any Teflon (left) and the finished pan with the Teflon applied (right), according to a proprietary process. The test for the TEP/lignin resin needed to be capable of withstanding more than 410 °C for 11 min under many repeated temperature applications. The bio-lignin/TEP assembly passed this repeated test well. Contrary to the tannin/TEP resin, the lignin/TEP resin system is not patented [32]. The reaction appears also for lignin, as for the tannin, to be dependent on the temperature. Thus, it is certainly favored from a 180 °C temperature, yielding insoluble hardened resins, as well as when ammonia is present, due to additional cross-linking reactions.

## 4. Totally Bio-Sourced Non-Isocyanate Polyurethanes (NIPU)

Polyurethanes, incorrectly called biopolyurethanes, can and have already been prepared by the use of bio-sourced polyols issued from renewable materials and used for a number of possible applications [34,35,36,37,38,39,40,41]. However, polymeric isocyanates have always been used to prepare such polyurethanes even if reacted with bio-sourced polyols. However, this approach can hardly be considered to lead to totally bio-sourced polyurethanes, seeing that toxic isocyanates are still used. Research works on alternate reaction routes yielding non-isocyanate-based polyurethanes (NIPUs) have now been known for a few years. These, however, are not bio-sourced and especially not bio-sourced starting from lignocellulosic materials. However, as good as these polyhydroxyurethanes [42,43,44,45,46,47,48,49,50,51,52,53,54,55,56,57,58,59] may be, they have shown to suffer from a few application drawbacks inherent to the approaches used, and this is on top of the lack of the use of bio-sourced renewable materials. The reaction is based on the reaction of single or double five-membered cycle carbonate groups [42] reacted with diamines [43,44,45,46,47,48,49,50,51,52,53,54,55,56,57,58,59]. The main technological barriers to their use are the need for the synthesis of single or double cyclic carbonates, bio-based or not, and thus an additional reaction, as well as the particular slowness in curing due to the slow reaction of opening of the cycles of the carbonates.

**Figure 15:**
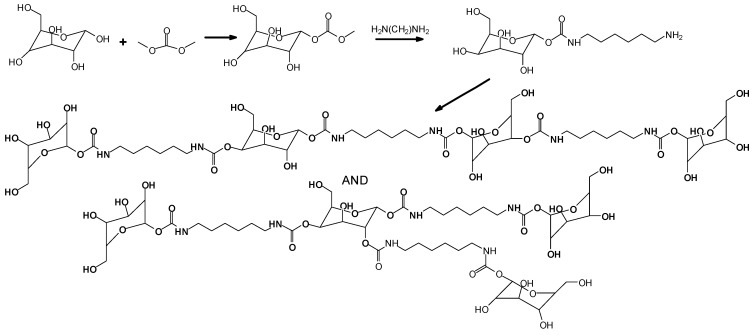
Linear and branched oligomers identified in glucose-based NIPUs [60].

**Figure 16:**
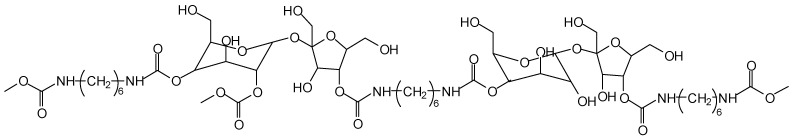
A polymeric species identified in sucrose-based NIPUs [62].

Non-isocyanate polyurethanes (NIPUs) that are totally bio-sourced have been obtained by reacting glucose and/or sucrose in a reaction with dimethyl carbonate, an inexpensive chemical, and hexamethylene diamine, also bio-sourced, or other bio-sourced diamine or polyamines [60] Dimethyl carbonate hydroxyl group carboxymethylation generally occurs at a temperature around 90 °C by bimolecular nucleophilic substitution, acyl-cleaving, under alkaline catalysis [61]. While this approach was initially successfully used for bio-sourced polyphenolic materials, these latter NIPUs have been tested on wood but have never been tested for coatings of metallic surfaces. The main objective of using glucose and sucrose was to demonstrate that NIPUs could be prepared from carbohydrates, a relatively inexpensive material in abundant supply. The oligomers produced in this manner were identified by MALDI-ToF, CP-MAS ^13^C NMR, and FTIR spectrometry. On steel, the NIPU resins were cured at 300 °C for 3 min, for both the glucose-based and sucrose-based coatings. The glucose derived NIPUs appeared to harden at a sensibly lower temperature and appeared to be easier to handle and spread than the ones prepared from sucrose due to the apparent glucose-based NIPU resin’s lower energy of activation of hardening. Steel coatings was, among others, the tested application of the glucose-based NIPUs [60] Cross-cut testing was carried out on the coated steel plates, and the results obtained were very encouraging. The coating results were already good at 103 °C for the glucose derived NIPU, while a markedly higher hardening temperature was needed for the sucrose-derived NIPU coating. Some of the higher-molecular-weight oligomeric NIPU species identified, both linear and branched, are shown in Figure 15 for glucose and in Figure 16 for sucrose.

**Figure 17:**
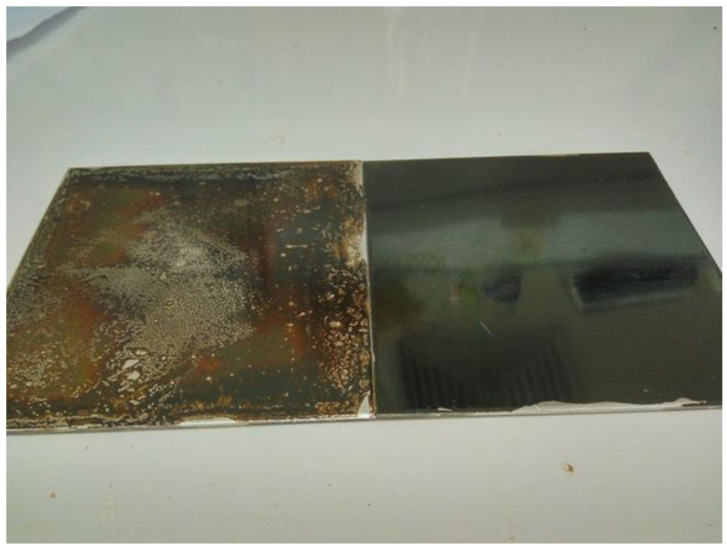
Appearance of glucose and sucrose NIPU coatings on steel. Left: sucrose NIPU coating. Right: glucose NIPU coating [61].

**Figure 18:**
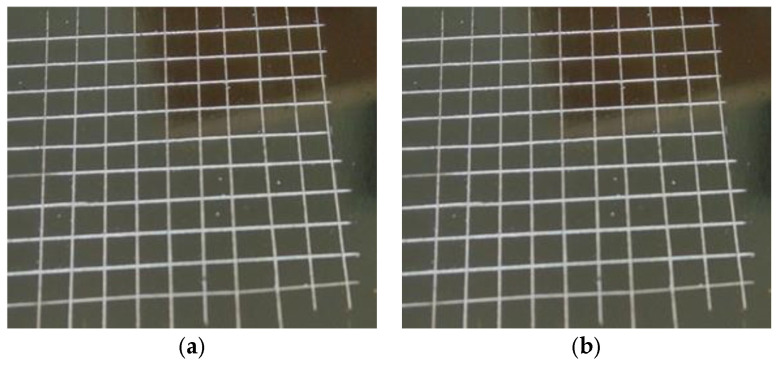
Cross-cut test according to [63] of a glucose-based surface coating on stainless steel cured for 3 min at 300 °C. (**a**) Before washing in hot water; (**b**) after washing in hot water [61].

The potential of this type of NIPU resin is shown by its capability of coating a steel surface. Figure 17 shows the hardened coating appearance of a steel plate with a glucose-derived NIPU resin and a sucrose NIPU resin. The clearly good appearance of the glucose derived coating film is apparent, its appearance also indicating this coating’s spreading ease. This was shown to be far better than the sucrose-derived NIPU coating [61]. The very bad appearance of the sucrose NIPU coating in Figure 17 indicates the very difficult spreading of such a coating due to the very high viscosity of this NIPU. Of particular interest is the cross-cut test results for the coating adhesion [61], which are shown in Figure 18, indicating that none of the cutting lattice squares were detached and showing that the cut line edges were smooth. Thus, the glucose-derived NIPU coating’s adhesion onto the metal plates was judged to be excellent.

## 5. PVA–Citric Acid Metal and Glass Bonding

Citric acid has in recent years gained considerable publicity as a wood binder, either alone or in combination with a variety of hydroxyl-carrying biomaterials, such as glucose, sucrose, starch, tannin, lignin, and others [64,65,66,67,68,69,70,71,72,73,74,75,76,77]. All this work has mainly aimed to the application of thermosetting wood adhesives, but a few research works exist in which some bio-adhesive systems have been used for adherence to metals or glass either as a coating or as an adhesive [78,79,80,81,82], in particular for glass [83,84,85,86].

A novel polyester-type citric acid/PVA adhesive (CPVA) was developed for plywood adhesive, but that was also able to bond steel and glass. It was accomplished by directly mixing the aqueous solution of PVA with citric acid, which was used to bond various substrates, including wood, but in particular glass, and steel sheets [87]. The adhesion strength of the adhesives was investigated by lap-shear tests in tension, using a universal testing machine under ambient conditions with a stretching speed of 1 mm/min. Taking glass slides as an example, the tested adhesive was compressed between two glass slides of 76 mm × 25 mm with a bonding area of 25 mm × 25 mm [87]. The results show that CPVA adhesives exhibit bonding properties on different substrates. Steel sheets, glass, and ceramics were selected for bonding, and lap-shear tests were performed. The bonding strength applied on glass and steel showed a clear trend, as shown in Table 1. The bonding strength of CPVA on both the glass and steel sheet was greater than 6 MPa, demonstrating a very strong bond being formed. Moreover, the bonding strengths of CPVA adhesives applied on glass and steel are both higher than that obtained with traditional polyurethane with soy protein and PVA/PAA adhesives. It appears that the CPVA adhesive has a greater potential for adhesion on glass and steel. Thus, CPVA could serve as a potential candidate for eco-friendly bonding on traditionally difficult-to-bond substrates.

The research work even compared the strength of the best PVA-citric acid adhesive with that of competitive commercial adhesives, showing that better results were obtained with this adhesive, as well as comparing the resistance of all of them to a warm water (63 °C) test, as shown in Table 2.

## 6. Conclusions

While the great majority of the rapidly increasing number of publications on lignocellulosic based (and other bio-sourced materials) adhesive and coatings are aimed for application to wood products bonding and wood surface finishing, there are relatively few works aimed at using lignocellulosic-derived bio-resins for bonding applications of, and coating on, metals, glass, and other difficult-to-bond substrates. While the natural tendency to use lignocellulosic materials for wood applications, the cases presented here have been shown to render readers aware that there are great opportunities to develop a variety of other higher-tech applications for lignocellulosic materials. Literally, the field is open to considerable and fast development once researchers start to become aware of the many unexplored possibilities that exist. This mini review has presented some unusual cases based on some lignocellulosic materials used for such non-traditional applications, showing encouraging potential for further considerable developments.

## Figures and Tables

**Table 1:** Dependence of the adhesion strength of glass and steel joints as a function of the ratio of citric acid/PVA (CA/PVA).

	CA:PVA Mass Ratio					
	0:1	0.25:1	0.5:1	0.75:1	1:1	1.1:1
Glass sheet (MPa)	5.2	5.75	6.0	6.1	6.4	5.9
Steel sheet (MPa)	4.7	5.5	5.7	5.8	6.3	5.9

**Table 2:** Strength results of steel joints bonded with different adhesives after a treatment in 63 °C water for 3 h.

Adhesive Type	MPa
PF	0.77
MUF	1.36
Modified Soy protein	1.07
Modified Starch	0.81
PVA-citric acid	1.52
